# *Encephalitozoon cuniculi* Infection of Domestic Rabbits (*Oryctolagus cuniculus*) in Slovenia between 2017 and 2021

**DOI:** 10.3390/pathogens12040516

**Published:** 2023-03-26

**Authors:** Maruša Škrbec, Alenka Dovč, Nina Mlakar Hrženjak, Brigita Slavec, Zoran Žlabravec, Nina Kočar, Olga Zorman Rojs, Jožko Račnik

**Affiliations:** Institute of Poultry, Birds, Small Mammals, and Reptiles, Faculty of Veterinary Medicine, University of Ljubljana, Gerbičeva 60, 1000 Ljubljana, Slovenianina.kocar@vf.uni-lj.si (N.K.);

**Keywords:** *Encephalitozoon cuniculi*, encephalitozoonosis, clinical signs, serology

## Abstract

*Encephalitozoon cuniculi* is a microsporidial parasite that primarily infects domestic rabbits (*Oryctolagus cuniculus*). It is the causative agent of encephalitozoonosis, a disease with an internationally recognized seroprevalence among rabbits. This study determines the presence, clinical manifestation, and serological status of encephalitozoonosis in pet rabbits in Slovenia using various diagnostic procedures. From 2017 to 2021, 224 pet rabbit sera were collected and tested for encephalitozoonosis with the indirect immunofluorescence assay. Immunoglobulin M (IgM) and immunoglobulin G (IgG) antibodies against *E. cuniculi* were confirmed in 160 (65.6%) cases. Most seropositive rabbits suffered from neurological clinical signs or signs of gastrointestinal disorders such as recurrent hypomotilities, chronic weight loss, cachexia, or anorexia, and fewer showed clinical signs related to the urinary system or phacoclastic uveitis. A quarter of the positively tested rabbits presented without clinical signs. Hematological and biochemical blood analysis confirmed that seropositive animals had elevated globulin and deviated albumin levels in comparison to the normal reference values of non-infected animals. Furthermore, rabbits with neurological clinical signs showed statistically significant higher levels of globulins and total protein. Sixty-eight whole-body radiographs and thirty-two abdominal ultrasound reports were analyzed, looking for changes in the shape or size of the urinary bladder, presence of urinary sludge or uroliths, and any abnormalities related to the kidneys (shape, size, or nephrolites). The results suggest that neurological defects in the urinary bladder caused by *E. cuniculi* lead to a distended urinary bladder and consequently dysuria, incontinence, urine scalding, and sludgy urine.

## 1. Introduction

*Encephalitozoon cuniculi* (*E. cuniculi*) is a single-celled intracellular pathogen related to fungi and classified in the phylum Microsporidia [1]. It is the causative agent of encephalitozoonosis, a widespread and significant disease of many domestic and wild mammalian animal species and even birds [2]. *E. cuniculi* is now also considered an opportunistic pathogen in immunocompromised people (AIDS patients, cancer patients undergoing chemotherapy, the elderly and children, diabetic patients, and travelers) causing multiple syndromes, such as encephalitis, keratoconjunctivitis, nephritis, hepatitis, myositis, peritonitis, sinusitis, and pneumonia [3]. Additionally, the zoonotic risk for people working with animals or their products and cleaning the animals’ cages must not be ignored, due to the close and continued physical contact with potentially infected animals and their urine. The parasite has a wide host distribution but primarily affects domestic rabbits (*Oryctolagus cuniculus*), which are considered the main host [4,5,6]. The disease is widespread among laboratory, farm, and pet rabbits. According to some studies, serological testing shows high seroprevalence rates (35–68%) across several countries [3,7,8]. These results suggest that *E. cuniculi* infection is ubiquitous in rabbits [9,10].

In rabbits, the central nervous system, kidneys, and eyes are usually predilection sites of infection with *E. cuniculi* [3]. However, other organs and tissues, such as the liver, gastrointestinal tract, and bones, can be affected [11,12]. Even though some infected rabbits may never show any signs of disease, other rabbits may display various clinical signs such as signs of renal failure, nephroliths, uroliths, eye lesions, neurological signs, and recurrent gastrointestinal hypomotility, or even sudden death [13,14,15]. 

The protective immunity following infection with *E. cuniculi* is mediated mainly through cell-mediated responses involving CD8+T and CD4+ cells, and to a lesser degree through humoral immunity. There is typically an increase in pathogen-specific immunoglobulin M (IgM) from day 0 to day 35 post-exposure and an increase in immunoglobulin G (IgG) at 2 or 3 weeks after exposure [2]. The IgG antibodies may decline or persist for years. Hence, the presence of IgG antibodies against *E. cuniculi* indicates past exposure to the parasite, without any indication of the timing of infection. However, high levels of IgM can be observed in very early infection (less than 30 days after infection), reactivated infection or reinfection. High levels of both IgG and IgM suggest an acute recent infection, while high levels of IgG with non-existing levels of IgM suggest past exposure to *E. cuniculi* [11,16]. Rabbits with the latter serological status are usually found clinically healthy but are at risk that stress or another suppressive factor may act as a trigger for encephalitozoonosis reactivation and therefore need to be monitored regularly for the presence of IgM antibodies. The immune response is not effective at completely clearing the pathogen, probably due to the ability of *E. cuniculi* to evade the immune response of the host [2].

Whereas clinically manifested neurological signs can be easily recognized, the in vivo diagnosis of encephalitozoonosis is often difficult because of the high percentage of subclinically infected animals [3]. In live rabbits, the diagnosis is usually achieved with a combination of clinical examination, ruling out possible differential diagnoses, and serological verification. Serological testing is the most widely used diagnostic method for *E. cuniculi* infection, even though the presence of antibodies does not confirm the organism as a causative agent of disease. Positive results can indicate chronic *E. cuniculi* infection [17].

To date, the status and prevalence of *E. cuniculi* infection in the pet rabbit population in Slovenia is unknown. In this article, the results of the data analysis of (suspected and diagnosed) *E. cuniculi* infections of domestic rabbits in Slovenia between 2017 and 2021 are presented. The main purpose was to obtain an overview of the clinical presentation and serological status of *E. cuniculi* infection in Slovenia. Furthermore, the results of various diagnostic procedures (particularly indirect immunofluorescence assay (IFA), blood analysis, radiographic imaging, or ultrasound examination) were used, and their effectiveness was rated for further information. The information gathered should aid clinicians through easier and better evaluation of clinical signs (in possible combination with other diagnostic procedures) to determine whether a rabbit could have encephalitozoonosis.

## 2. Materials and Methods

### 2.1. Animals

The data from 224 pet rabbits were included in a retrospective study of encephalitozoonosis in Slovenia. All rabbits were companion animals, living in the same household as their owners. The majority of the rabbits were mixed breeds, weighed approximately 2 kilograms, and were between 5 months and 10.5 years old, with a mean age of 4.98 years. The animals were presented to the University Clinic of Birds, Small Animals, and Reptiles (Veterinary Faculty, Ljubljana, Slovenia) between January 2017 and July 2021. The animals were either brought to the clinic due to illness or for preventive clinical examination and testing for encephalitozoonosis. All the admitted rabbits were tested for *E. cuniculi* infection with the IFA. If needed, other diagnostic methods to assess their general health condition were also performed, such as blood analysis, radiography, and abdominal ultrasound.

Information about different types of clinical signs, laboratory results from hematological and biochemical assays, ultrasound reports and radiographic image analyses of animals that tested positive (*n* = 160), meaning they had specific immunoglobulin M (IgM) or immunoglobulin G (IgG) antibodies against *E. cuniculi*, were used for further statistical analysis. The positive population was our main study subject of interest. Animals that tested negative (*n* = 64) were not included in further statistical analyses or division into clinical signs groups because encephalitozoonosis was not considered the main cause of their clinical condition.

### 2.2. Clinical Examination

All studied rabbits that were presented to the clinic underwent a prompt clinical examination. Depending on their clinical status, seropositive animals (*n* = 160) were divided into five groups. Group 1 (clinically healthy) was specified as the preventive health check group. Animals in this group showed no obvious clinical signs but had elevated IgM and/or IgG. Group 2 (neurological signs) consisted of animals with neurological clinical signs including vestibular disease (head tilt, nystagmus, circling, rolling, or imbalance), tremors, or weakness of hind legs. In Group 3 (urinary system signs), signs of urinary tract disorders were seen, including polyuria, polydipsia, dehydration, anorexia, weight loss, sludgy urine, urine scalding, and difficulty urinating. Group 4 (eye disorder signs) consisted of rabbits with signs of phacoclastic uveitis (showing as cataract, uveitis, or granuloma in the eyes). Group 5 (gastrointestinal signs) included rabbits with recurrent gastrointestinal hypomotilities, cachexia, anorexia, and chronic weight loss that could have been caused by a chronic *E. cuniculi* infection.

### 2.3. Indirect Immunofluorescence Assay

With an intravenous catheter, blood was collected from the marginal ear vein of all 224 rabbits. BD Microtainer tubes containing Clot Activator / SST™ Gel were used for collecting blood (Becton, Dickinson and Company, Franklin Lakes, NJ, USA). After centrifugation at 2000 rpm for 10 min, sera were collected and tested for the presence of specific antibodies against *E. cuniculi*. Serological diagnosis was carried out using IFA, using the commercial test-kit (MegaFLUO®, ENCEPHALITOZOON cuniculi, Megacor, Austria). Anti-rabbit IgM and IgG antibodies, conjugated with Fluorescein-Iso-Tio-Cyanate (FITC), were added (Megacor, Austria). Each serum was tested and titrated from 1:40 to 1:1280. Conjugates were added according to manufacturers’ instructions. 

### 2.4. Hematological and Biochemical Assays

Blood samples from seropositive rabbits that needed additional diagnostics to assess their general health were tested at the laboratory of the Small Animal Clinic of the Veterinary Faculty, University of Ljubljana, for a hematological and biochemical analysis. For both the hematological and biochemical assays, the individual blood samples were collected in BD Microtainer blood collection tubes containing lithium heparin as an anticoagulant (Becton, Dickinson and Company, Franklin Lakes, NJ, USA). Since the proceeding of diagnostic procedures on the animals depended on the owner’s permission and their financial limitations, unfortunately not all seropositive rabbits had their blood samples taken and sent to the laboratory for hematological and biochemical assays. Consequently, the number of animals for which hematological and biochemical data were available varied.

For hematological analyses, the blood samples of 96 *E. cuniculi*-positive rabbits were analyzed using an automated laser-based ADVIA 120 hematology analyzer with a species-specific setting for rabbits in the multi-species software developed by the manufacturer of the analyzer (Siemens, Munich, Germany). Factory software settings were used without adjustments or modifications. The analyzer utilizes the principle of automated cytochemistry coupled with flow cytometry. The hematological parameters included white blood cell (WBC) count, red blood cell (RBC) count, platelet count (PLT), procalcitonin (PCT), number of neutrophils (NEUT), lymphocytes (LYMPH), monocytes (MONO), eosinophils (EOS), basophils (BASO), large unstained cells (LUC), hematocrit (HCT), and hemoglobin (HGB). The distribution of hematological assays between the groups of clinical signs was as follows: 17 hematological analyses from clinically healthy animals, 42 hematological analyses from rabbits with neurological clinical signs, 9 hematological analyses from rabbits with urinary system signs, 28 hematological analyses from rabbits with gastrointestinal signs and zero hematological analysis from rabbits with eye disorder signs.

Individual blood samples of 113 *E. cuniculi*-positive rabbits underwent a biochemical analysis with the portable VetScan® VS2 Chemistry Analyzer (Zoetis, Union City, CA, USA). VetScan Comprehensive Diagnostic Profile Reagent or (mainly) VetScan Equine Profile Plus reagent rotors (both Abaxis, Inc., Union City, CA, USA) were used. For the statistical analyses, we focused on the following parameters: albumins (ALB), globulins (GLOB), alkaline phosphatase (ALP), alanine aminotransferase (ALT), amylase (AMY), aspartate aminotransferase (AST), blood urea nitrogen (BUN), calcium (Ca), sodium (Na), creatine kinase (CK), creatinine (CRE), gamma-glutamyl transferase (GGT), glucose (GLU), potassium (K), inorganic phosphorus (PHOS), total protein (TP), and total bilirubin (TBIL). The distribution of biochemical assays between the groups of clinical signs was as follows: 19 biochemical analyses from clinically healthy animals, 49 biochemical analyses from rabbits with neurological clinical signs, 10 biochemical analyses from rabbits with urinary system signs, 34 biochemical analyses from rabbits with gastrointestinal signs and only 1 biochemical analysis from rabbits with eye disorder signs.

### 2.5. Other Diagnostic Procedures

If additional diagnostic procedures were necessary, individual seropositive rabbits underwent other diagnostic tests, such as abdominal radiography (*n* = 68) and ultrasound (*n* = 32). Distribution of abdominal ultrasound reports between the groups of clinical signs was as follows: 6 reports from clinically healthy animals, 14 reports from rabbits with neurological clinical signs, 4 reports from rabbits with urinary system signs, 8 reports from rabbits with gastrointestinal signs and none reports from rabbits with eye disorder signs. Distribution of radiographic images among the clinical signs groups was as follows: 8 radiographs from clinically healthy rabbits, 27 radiographs from rabbits with neurological clinical signs, 7 radiographs from animals with urinary system signs, 24 radiographs from rabbits with gastrointestinal signs and only 2 radiographic images from rabbits with eye disorder signs. For abdominal ultrasound, the Logiq S7 Expert/Pro (GE Ultrasound Korea, Ltd., Seongnam, South Korea) machine was used. Abdominal examination was performed with the 11L linear ultrasound probe. For the radiographic analysis and measurements of urinary bladder length (mm), Vet-Exam plus 7.3.0 Imaging Software (2012, DÜRR NDT GmbH & Co KG, Bietigheim-Bissingen, Germany) was used.

## 3. Results

### 3.1. Clinical Examination

Out of all 224 rabbit cases included in the study, the owners of 169 animals (75.4%) made a veterinary appointment because they had noticed signs of illness in their animals. The other 55 rabbits (24.6%) were tested for encephalitozoonosis at the owners’ request and were classified as overall healthy animals. With IFA, 160 (71.4%) rabbits tested positive for the presence of either IgM and/or IgG antibodies against *E. cuniculi*. Of these 160 seropositive animals, 40 (25.0%) were non-clinical for any type of encephalitozoonosis-linked signs and were classified into Group 1 (clinically healthy). The other 120 seropositive rabbits (75.0%) showed various kinds of clinical signs, upon which they were divided into four further groups. Animals with central vestibular disease including clinical signs such as torticollis with head-tilt, nystagmus, imbalance, rolling or leaning to one side, or weakness of the hind legs represented 62 (38.8%) positive-tested rabbits and were classified into Group 2 (neurological signs, *n* = 62). Rabbits showing clinical signs associated with urinary tract disorders represented 7.5% (*n* = 12) of positive-tested animals and were assigned to Group 3 (urinary system signs). Usually, clinical presentation of polyuria/polydipsia, dehydration, anorexia, weight loss, sludgy urine, urine scald, and difficulty urinating were observed. Signs of phacoclastic uveitis was observed in three seropositive rabbits (1.9%) and was therefore the least represented sign of encephalitozoonosis. These rabbits were categorized in Group 4 (eye disorder signs). Finally, clinical signs of gastrointestinal disorders (recurrent hypomotilities, chronic weight loss, cachexia, or anorexia) were observed in 43 seropositive rabbits (26.9%), which were categorized into Group 5 (gastrointestinal signs).

In the table below (Table 1), a display of the studied rabbit groups is presented. Seropositive rabbits in the clinical signs groups from 1 to 5 are numerically defined. Seronegative rabbits were not included in the clinical signs groups, because encephalitozoonosis was not considered the main cause of their clinical condition.

Out of all IFA-positive animals tested for the presence of *E. cuniculi*, 65 female and 95 male rabbits were positive. We examined the relationship between age and infection with *E. cuniculi*. The relationship was tested using the χ² test. The results show that the association between age and infection with *E. cuniculi* is not statistically significant (χ^2^ (1) = 0.42, *p* = 0.518). We also tested the relationship between infection with *E. cuniculi* and the sex of the animal. Similarly, the results were not statistically significant (χ^2^ (1) = 0.38, *p* = 0.537).

### 3.2. Indirect Immunofluorescence Assay

With the IFA, we were able to gather information regarding previous *E. cuniculi* exposure. Out of 224 rabbits, 64 animals tested negative, meaning that both IgM- and IgG-specific antibodies against *E. cuniculi* were absent (IgM−, IgG−). Among all the animals that tested positive (*n* = 160), the results of their serological status are the following. Rabbits with both IgM and IgG antibodies (IgM+, IgG+; *n* = 119) comprised 74.4% of all animals that tested positive. Thirty-six (*n* = 36, 22.5%) animals only had specific IgG antibodies elevated (IgM−, IgG+), whereas a smaller number of rabbits (*n* = 5) tested positive for only IgM antibodies (IgM+, IgG-), which is 3.1% of the animals that tested positive. In all five groups, the number of rabbits with the combination of elevated IgM and IgG specific antibodies against *E. cuniculi* was the highest, whereas the presence of only IgM antibodies was the lowest or even absent in some groups.

Information about the serological status of rabbits in individual groups is presented in Table 2.

### 3.3. Hematological and Biochemical Analyses

Out of the seropositive group, blood samples from 96 and 113 rabbits were available for hematological and biochemical analyses, respectively, to investigate possible dissimilarities between the rabbits in the five different groups. The differences in hematological parameters between groups were tested using the Kruskal–Wallis test. The results show that the differences in the distribution of hematological parameters were not statistically significant between groups. The same test was also performed for the biochemical blood parameters between the five groups. These results show that the differences in the distribution of globulins (GLOB) and total protein (TP) between groups were statistically significant (*p* < 0.05). Furthermore, cases with neurological signs (Group 2) had higher medians of globulins and total protein compared to other groups. Due to missing values or insufficient sample size, we did not include the following parameters: ALT, AST, ALP, AMY, CK, PHOS, and GGT.

**Figure 1:**
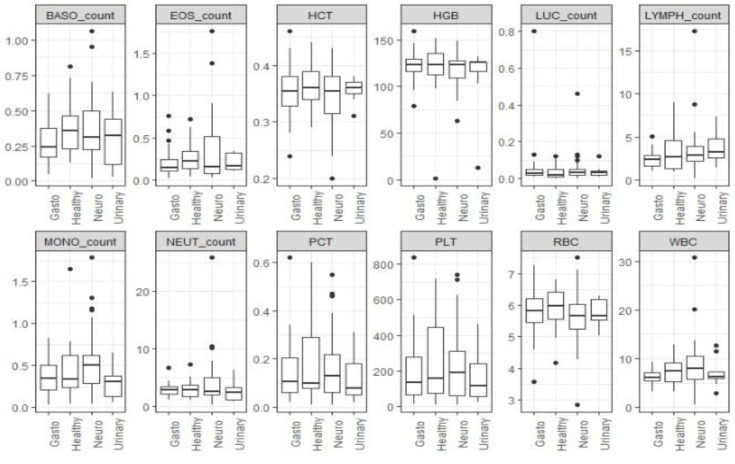
Boxplot of hematological parameters for four groups (with different clinical signs): Group 1 (clinically healthy), Group 2 (neurological clinical signs), Group 3 (urinary system signs), and Group 5 (gastrointestinal signs). Group 4 (eye disorder signs) was not included in the boxplot because only one biochemical assay result was available.

**Figure 2:**
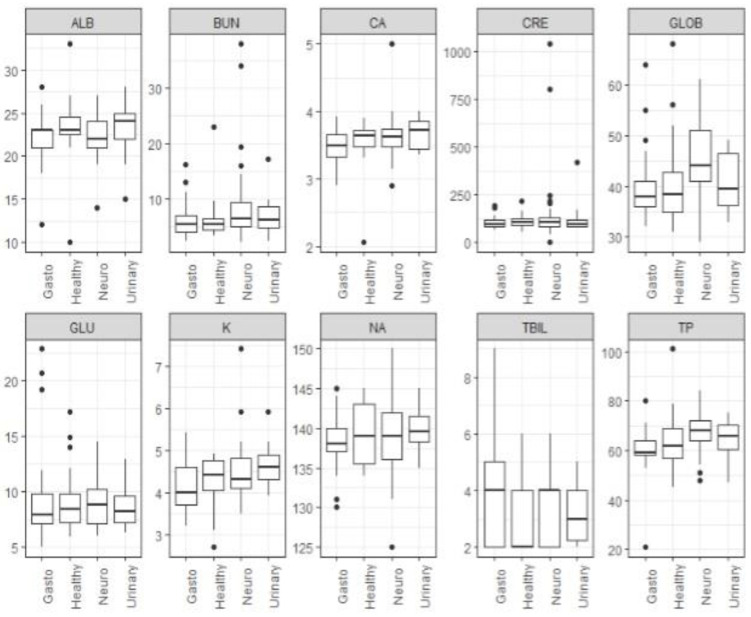
Boxplot of biochemical parameters for four groups (with different clinical signs): Group 1 (clinically healthy), Group 2 (neurological clinical signs), Group 3 (urinary system signs), and Group 5 (gastrointestinal signs). Group 4 (eye disorder signs) was not included in the boxplot because only one biochemical assay result was available.

Boxplots of hematological parameters (Figure 1) and biochemical parameters (Figure 2) for clinical signs groups are presented below.

In addition, we tested whether the median values of the hematological or biochemical parameters for rabbits infected with *E. cuniculi* were statistically different compared to the upper and lower reference values for healthy rabbits. Reference values for healthy rabbits were taken from *Exotic Animal Formulary* (4th edition) by James W. Carpenter [18] and the values set by the automated laser-based ADVIA 120 hematology analyzer. We calculated the confidence intervals for the median value for each parameter. The results show that there is no overlap between confidence intervals for the median values and reference intervals for the variables GLOB and ALB. More detailed results are listed in Table 3.

### 3.4. Other Diagnostic Procedures

Abdominal radiographs of 68 rabbits with confirmed encephalitozoonosis were available during our data research. Radiographic images in the latero–lateral and ventro–dorsal view were used. Images of rabbits with neurological clinical signs (*n* = 27) or urinary system disorders (*n* = 7) were examined especially closely for any changes in the size of the urinary bladder, the presence of urinary sludge or uroliths, or any abnormalities related to the kidneys (abnormal shape, size, or nephroliths).

Of all the rabbits, no animal had any obvious alterations of the kidneys seen on the radiographs. In addition, no nephroliths or uroliths were discovered. Four rabbits (14.8%) with neurological clinical signs had a moderate to large amount of urinary sludge present. Twelve rabbits (44.4%) with neurological clinical signs had a lightly to moderately enlarged/distended urinary bladder. In rabbits with urinary system disorder, the presence of urinary sludge was stronger (*n* = 5/7, 71.4%). In addition, all five animals with urinary sludge simultaneously had a largely distended urinary bladder. The measured lengths of the urinary bladder of rabbits with neurological or urinary system signs are listed in Table 4.

The abdominal ultrasound examination reports of 32 *E. cuniculi*-positive rabbits were available. The main focus of this diagnostic method was to examine the size, shape, and structure of both kidneys and to determine the presence of urinary sludge, nephroliths, or uroliths. As a size reference for the left and right rabbit kidney (1.1–2.5 kg), the measurements from Banzato et al. were used [19]. Depending on the article, the ultrasound reference values for the mean size of the right kidney are 2.87 ± 0.34 cm for the length and 1.62 ± 0.17 cm for the width. Reference values for the mean size of the left kidney are 2.86 ± 0.33 cm for the length and 1.72 ± 0.19 cm for the width. In our study, the calculated mean length of 22 rabbits’ right kidneys was 2.77 cm and the width 1.77 cm, whereas the mean length of the left kidney was 2.88 cm and width 1.85 cm. The given mean sizes of both kidneys were slightly higher than the reference sizes.

## 4. Discussion

This study examines encephalitozoonosis among Slovenian rabbits, showing that its clinical presentation is quite variable. In *E. cuniculi* IgM and/or IgG serological-positive rabbits (*n* = 160), there was a strong presence of both neurological clinical signs (38.8%) and, as now registered as nonspecific disease presentations, gastrointestinal disorders (27.5%), such as recurrent hypomotilities, chronic weight loss, cachexia, or anorexia. In the setting of an active *E. cuniculi* infection, focal granulomatous infiltrates can be histologically observed in various organs and tissues, also in the intestines [20]. The effects of granulomatous infections in two of the most commonly affected systems (kidneys (*n* = 12) and central nervous system (*n* = 62)) can cause nausea, apathy, and anorexia, and consequently development of gut hypomotility [14]. Furthermore, rabbits are particularly susceptible to the effects of stress, which is certainly present during an ongoing disease. In rabbits, adrenaline can cause a marked and prolonged reduction in renal plasma flow, and gastric ulcers are associated with stress. Stress can have a dramatic effect on digestive function. Stimulation of the sympathetic nervous system inhibits gut motility [15,21]. We believe that these reasons could be the cause of gastrointestinal disorder due to encephalitozoonosis. Also, in the author’s experiences, *E. cuniculi*-seropositive rabbits that present to the clinic with repetitive gastrointestinal hypomotility with no other identified problems respond well to treatment with fenbendazole (20 mg/kg, orally, once a day for 28 days) and metamizole (65 mg/kg, orally, twice daily for two weeks). After the treatment, the frequency of hypomotilities successfully decreases.

Certainly, it is important to rule out other causes that could lead to gastrointestinal or other *E. cuniculi*-related disorders, such as stomatology problems, other bacterial, viral, or parasitic pathogens (*Pasteurella multocida*, *Toxoplasma gondii*, *Eimeria* spp.), improper diet, and so on [15,22]. A clinician should always consider other causalities for the clinical signs and decide for a suitable diagnostic method based on the rabbits’ clinical presentation, a comprehensive medical history of the animal, and the owners’ anamnesis. A prompt clinical examination of all seropositive rabbits should be done, and detailed anamnesis always taken from the owners. For example, with a good anamnesis, a clinician can easily eliminate the possibility of a trauma event that could cause neurological clinical signs, teeth damage, and consequently gastrointestinal problems. Furthermore, co-housing the rabbit with a cat or feeding the rabbit with grass or vegetables from the garden could result in *Toxoplasma gondii* infection that would show some similar neurological clinical signs as *E. cuniculi* infection. Any changes in the rabbits’ diet, consummation of toxins or other stressful events in the animals’ life can also lead to gastrointestinal problems. After gathering information about these topics, some differential diagnosis mentioned above can be eliminated. With a careful clinical examination, the veterinarian can once again exclude an ear infection as the causative for neurological signs, stomatology problems or fur shedding that could lead to hypomotilities, and any (nasal) discharges that could be present in, for instance, an infection with *Pasteurella multocida*. With the microscopic examination of feces, *Eimeria* spp. can easily be ruled out as a causative of gastrointestinal disorders, apathy, dehydration, or lethargy. After the conversation with the owner and clinical examination of the rabbit, the clinician should have gathered enough information about the animals’ clinical status to see whether there is still a chance for an *E. cuniculi* infection. Further diagnostic methods, such as ultrasound examination of the abdomen to eliminate any (tumorous) growths on the organs and any obvious pathology or inflammation of the urinary bladder, should be used. Radiographic images of the body in the latero–lateral and ventro–dorsal view are advised to be taken to exclude any pathological findings on the spine or other bone structures that could cause neurological clinical signs or chronic pain and stress related to it. Radiographic images of the rabbits’ skull could give us an insight into possible stomatology problems, or any tympanic bullae pathology, which can cause vestibular signs (e.g., ataxia, nystagmus, or head tilt) [23]. All of our rabbit study population individually underwent the needed diagnostic procedures to eliminate other differential diagnosis that could cause their clinical signs. Despite of the best effort to discover it, there is always a slight chance that besides *E. cuniculi* some other disguised health condition is ongoing in the animals’ body. 

Signs of phacoclastic uveitis (1.9%) and especially urinary tract problems (7.5%) were not as strongly represented clinical signs among our tested rabbit population in comparison to the number of rabbits suffering from other clinical signs in the study. Similar to our findings, in other studies the eye disorder signs and urinary system signs were also less represented in the rabbit study population than, for example, neurological clinical signs [3,24,25]. There was also a high percentage of clinically healthy rabbits (Group 1) with confirmed *E. cuniculi* infection (25.0%) with none of the clinical signs mentioned in this study. These animals were mainly brought to the clinic for testing by owners, who are aware of encephalitozoonosis and its risks. This might show that there is a rising awareness of *E. cuniculi* among owners of pet rabbits, which is of great importance because it is a potential zoonosis [26]. The information about the serological status is primarily also beneficial for the veterinarian treating the affected rabbit.

This study confirmed that the rabbit’s age and sex had no significant effect on *E. cuniculi* infection, which is in agreement with studies performed on domestic rabbits [2,11,27].

Simultaneous testing of both IgM and IgG offers an indication of the animal’s infection status. Whereas IgG antibodies against *E. cuniculi* are an indicator of past exposure to *E. cuniculi* or resolved infection, the specific IgM antibodies reflect an acute, reactivated infection or reinfection [11,22]. The combination of both IgM and IgG means that the current ongoing clinical signs are a consequence of active encephalitozoonosis. An active infection was detected in considerably high numbers of rabbits among every group of clinical signs, especially in the group of neurologic clinical signs and urinary system disorders (see Table 2). This finding was not surprising because both groups of clinical signs in rabbits are the most common for encephalitozoonosis, and for a veterinary practitioner they are relatively easy to detect during a clinical examination, raising the suspicion of infection [2,10]. In addition, a fair number of rabbits without any clinical signs also had IgM anti-*E. cuniculi* antibodies present. The detection of IgM antibodies in combination with clinical signs of encephalitozoonosis requires administration of the proper antimicrosporidial therapy [28]. Therefore, the detection of both isotypes of specific antibodies should be considered a routine part of a health check in rabbits. Information about a rabbit’s serological status is important not only from the veterinary perspective but also for the rabbit’s owners who are at greater risk of contracting encephalitozoonosis, such as immunocompromised people, children, and the elderly [7]. For further research, it would be interesting to determine whether there is a positive correlation between specific *E. cuniculi* antibody titers and the severity of the clinical signs in positive rabbits.

Hematological and biochemical analysis was carried out to compare different parameters between the five groups of animals and to compare the parameters of *E. cuniculi*--positive animals to the reference values of healthy rabbits. The results showed that rabbits with neurological signs (Group 2) had statistically significant higher levels of globulins and total protein in comparison to other clinical signs/groups. Similar, rabbits with *E. cuniculi* infection showed elevated globulin and deviated albumin levels in comparison to normal upper and lower reference values of non-infected animals. These results were not unexpected since some previous studies [14,29] showed that rabbits with encephalitozoonosis do have higher gamma globulin and lower albumin levels than clinically normal rabbits. Albumin is a “negative acute phase protein” that is expected to decrease in the acute phase of certain inflammatory processes [14]. In addition, even though *E. cuniculi* infections may appear clinically acute, they are typically a chronic inflammatory disease process; hence a rise in gamma globulins is expected in active cases [14]. In our study no significant differences were observed in terms of other parameters between *E. cuniculi* serum samples and referral values of healthy animals or between the same parameters in different groups. Even though it was expected that Group 3 animals (urinary system signs) would have higher parameters connected to urinary tract problems, such as creatinine (CREA), urea (BUN), phosphorus (P), sodium (Na), potassium (K), and so on, their levels surprisingly exhibited non-significant changes when compared to the same parameters in other groups or values from healthy rabbits. The reason for these results probably lies in the small number of animals with renal clinical signs in the study. On the other hand, previous studies show that elevations in serum/plasma creatinine can occasionally be noted in rabbits with encephalitozoonosis, but these findings are not reliable indicators of *E. cuniculi* infections [13,14].

With the ultrasound examination (*n* = 32) and radiographic images (*n* = 68), we mainly focused on finding any potential changes in the size of the urinary bladder or the presence of urinary sludge, uroliths, or abnormalities related to the kidneys (abnormal shape, size, nephroliths, etc.). Radiographs in fact did not show any obvious alterations of the kidneys or the presence of nephroliths or uroliths. Some interesting results were found regarding active encephalitozoonosis serological status and changes to the urinary bladder. To date, there have been no studies investigating the direct clinical impact of *E. cuniculi* on the urinary bladder size or distension in rabbits. Namely, it is suspected that encephalitozoonosis is linked to formation of sludge, incontinence, and dysuria among other kidney-related pathologies [14,30]. No articles describe the presence of distended urinary bladder as a side effect of encephalitozoonosis. We suspect that *E. cuniculi*-associated neurologic effects could disturb the normal urinary bladder function/contractions and consequently distend the urinary bladder wall because urine is not passed normally. Unfortunately, no reference values of a normal and healthy rabbit urinary bladder size or volume to compare with our measurements were found in the literature. Consequently, we used the calculated urinary bladder mean size from the group of clinically healthy and *E. cuniculi*-negative rabbits included in our study. The calculated mean length of 11 urinary bladders was 32.2 mm. The mean weight of the animals was 2.3 kg. Urinary bladders that were larger than the calculated control group mean size were perceived as enlarged/distended, especially if the rabbit was suffering from dysuria, incontinence, urine scalding, and sludge urine. Even though our study population of *E. cuniculi*-positive rabbits with largely distended urinary bladders and clinical signs associated with this was quite small, serologically all the animals had an active form of encephalitozoonosis. Certainly, other neurological and urinary tract diseases can also lead to incontinence, difficulty urinating, and consequently urinary retention in the urinary bladder. A veterinarian must always consider this possibility and decide for a suitable diagnostic method based on the rabbits’ clinical signs, medical history, and the owners’ anamnesis to rule out other causes for the observed clinical presentation. Further studies about a possible link between encephalitozoonosis and its effects on the urinary system should be undertaken to determine whether our theory about bladder enlargement could be of any significance. A pathological investigation of deceased seropositive rabbits that suffered from urinary system signs to obtain additional information about the urinary bladder status would be useful as well.

Abdominal ultrasound reports showed some more individually related brief changes to the urinary system. Out of 32 seropositive animals, a slight or moderate presence of urinary sludge was discovered in six. Serologically, four of them had an active form of encephalitozoonosis (IgM+, IgG+). One rabbit had nephroliths in the left kidney and a slightly enlarged right kidney. Its serological status showed active encephalitozoonosis (IgM+, IgG+). Finally, two rabbits had some renal pathology present (enlarged kidneys, calcifications in the renal cortex, and a poorly defined kidney corticomedullary boundary) but did not have elevated IgM antibodies (IgM−, IgG+). The number of available ultrasound reports and the study population are both unfortunately too small to draw any conclusions between the correlation with active ongoing encephalitozoonosis and the pathologies mentioned above. Further research should be dedicated to this topic. Regarding the correlation between the size of kidneys and encephalitozoonosis, some authors have reported that chronic interstitial nephritis, often associated with subclinical infections caused by *E. cuniculi*, could cause a reduction in kidney size [31,32]. In our study, this theory was not confirmed because the mean sizes of the right and left kidney length and height were even slightly higher than the reference we referred to. As a disclaimer, a potential slight chance of measurement error should be considered.

Some parameters and results of the diagnostic methods used in the study (elevated globulin and simultaneously deviated albumin levels, clinical signs of recurrent hypomotilities with other causalities ruled out, radiographic findings of an enlarged bladder in rabbits with dysuria, incontinence, and urinary sludge) may help the veterinary clinician to raise the suspicion of encephalitozoonosis. Still, simultaneous serological testing is needed to confirm *E. cuniculi* infection. For now, in Slovenia, the most common and reliable method is still the use of an indirect immunofluorescence assay. In the future, a more detailed observation plan and anamnesis during clinical examination could be developed, as well as a more advanced overview of some ultrasonographic or radiographic features. Abroad, the use of polymerase chain reaction (PCR), enzyme-linked immunosorbent assay (ELISA), and even Western blot is increasing [33]. The mentioned techniques are also available in Slovenia but are currently not yet validated as a diagnostic method for encephalitozonosis. Definitely, they could have some new potential in the future.

## 5. Conclusions

In conclusion, the findings of this present survey, the first performed in Slovenia, show that *E. cuniculi* is present in pet rabbits in Slovenia. The infected rabbits mostly suffer from neurological clinical signs and urinary tract disorders, but a high percentage of gastrointestinal disorders were also seen. More studies regarding rabbits with this clinical manifestation and pertaining serological status should be performed in the future. Some rabbits with active encephalitozoonosis that suffered from urinary tract disorders and neurological clinical signs had an enlarged urinary bladder, as seen on radiographs. We believe that there may be a positive correlation between the neurological defects caused by *E. cuniculi* and a distended urinary bladder that clinically manifests as dysuria, incontinence, urine scalding, and sludgy urine. A large number of positive rabbits had serologically elevated titers of IgM antibodies, which indicates an active infection, and therefore preventive serological testing for *E. cuniculi* in pet rabbits with or without clinical signs should be performed regularly. For clinicians, the criteria used to suspect encephalitozoonosis would be clarified as a rabbit showing neurological clinical signs, urinary system signs or gastrointestinal signs discussed in this study, with simultaneously elevated IgM and IgG antibody levels. Based on zoonotic potential for owners, knowledge about the serological status of pet rabbits is also of great importance. 

## Figures and Tables

**Table 1:** Clinical signs group division of *E. cuniculi* positive-tested rabbits (seropositive rabbits) into 5 groups.

	Seropositive Rabbits (*n* = 160)					Seronegative Rabbits (*n* = 64)
	**Group 1** (clinically healthy)	**Group 2** (neurological clinical signs)	**Group 3** (urinary system signs)	**Group 4** (eye disorder signs)	**Group 5** (gastro- intestinal signs)	/
***n***	40	62	12	3	43	/

**Table 2:** Serological status of rabbits within different groups of clinical signs of *E. cuniculi* infection.

Groups of Clinical Signs	Serological Status	*n*	%
Group 1 (Clinically healthy)	IgM+, IgG+ IgM−, IgG+ IgM+, IgG−	25/40 13/40 2/40	62.5 32.5 5.0
Group 2 (Neurological signs)	IgM+, IgG+ IgM−, IgG+ IgM+, IgG−	52/62 10/62 0/62	83.9 16.1 0
Group 3 (Urinary system signs)	IgM+, IgG+ IgM−, IgG+ IgM+, IgG−	11/12 1/12 0/12	91.7 8.3 0
Group 4 (Eye disorder signs)	IgM+, IgG+ IgM−, IgG+ IgM+, IgG−	2/3 0/3 1/3	66.7 0 33.3
Group 5 (Gastrointestinal signs)	IgM+, IgG+ IgM−, IgG+ IgM+, IgG−	29/43 12/43 2/43	67.4 27.9 4.7

**Table 3:** Median values of hematological and biochemical parameters for rabbits infected with *E. cuniculi* in comparison to upper and lower reference values for healthy rabbits.

Variable	Unit	*n*	Lower Reference	Upper Reference	Median [95% CI]
ALT	U/I	59	0.00	61.00	36 [30; 37]
AST	U/I	91	0.00	28.00	27 [24; 28]
ALP	U/I	59	0.00	397.00	43 [36; 48]
AMY	U/I	59	0.00	459.00	176 [149; 186]
BUN	mmol/L	152	2.1	8.4	5.6 [5; 5.65]
CRE	μmol/L	151	34.00	166.00	97 [91; 104]
CK	U/I	92	0.00	958.00	588 [508; 763]
GLU	mmol/L	151	4.2	8.3	8.3 [7.8; 8.8]
K	mmol/L	150	3.7	6.3	4.35 [4.2; 4.4]
PHOS	mmol/L	57	0.80	3.20	0.82 [0.78; 0.89]
CA	mmol/L	151	2.90	3.10	3.6 [3.51; 3.64]
NA	mmol/L	151	138.00	155.00	139 [138; 138]
GGT	U/I	92	0.00	13.00	9 [8; 9]
GLOB	g/L	150	15.00	35.00	40 [38; 40]
ALB	g/L	152	36.00	57.00	23 [22; 23]
TP	g/L	151	49.00	74.00	63 [60; 63]
TBIL	μmol/L	151	0.00	12.80	4 [3; 4]
WBC	×10^9^/L	122	2.90	8.10	6.84 [6.15; 7.2]
RBC	×10^12^/L	122	4.20	6.70	5.76 [5.64; 5.84]
PLT	×10^9^/L	122	136.60	558.40	137 [98.5; 160]
HGB	g/L	122	95.00	145.00	124 [120; 126]
HCT	L/L	122	0.27	0.46	0.36 [0.35; 0.36]
PCT	%	122	0.10	0.40	0.11 [0.0814; 0.12]
NEUT	×10^9^/L	122	0.8	2.9	2.78 [2.44; 3.02]
LYMPH	×10^9^/L	122	2.2	5.3	2.62 [2.4; 2.8]
MONO	×10^9^/L	122	0.00	0.4	0.4 [0.3; 0.46]
EOS	×10^9^/L	122	0.00	0.4	0.18 [0.14; 0.22]
BASO	×10^9^/L	122	0.1	0.6	0.29 [0.25; 0.32]
LUC	×10^9^/L	122	0.00	0.04	0.03 [0.02; 0.03]

**Table 4:** Radiographic measurements of the urinary bladder length of rabbits with either neurological or urinary system signs.

Group	Length of Urinary Bladder (mm)	Mean Weight (kg)
Group 2 (Neurological clinical signs)	34 36 40 45 45 45 50 50 51 53 55 56	2.2
Group 3 (Urinary system signs)	60 66 74 82 94	2.3

## Data Availability

The data presented in this study are available on request from the corresponding author. The data are not publicly available due to client’s data privacy protection.
